# Nasopharyngeal Swabs Are More Sensitive Than Oropharyngeal Swabs for COVID-19 Diagnosis and Monitoring the SARS-CoV-2 Load

**DOI:** 10.3389/fmed.2020.00334

**Published:** 2020-06-18

**Authors:** Huan Wang, Qian Liu, Jing Hu, Min Zhou, Mu-qing Yu, Kai-yan Li, Dong Xu, Yao Xiao, Jun-yi Yang, Yan-jun Lu, Feng Wang, Ping Yin, Shu-yun Xu

**Affiliations:** ^1^Department of Orthopedic Surgery, Tongji Hospital, Tongji Medical College, Huazhong University of Science and Technology, Wuhan, China; ^2^Department of Respiratory and Critical Care Medicine, Key Laboratory of Pulmonary Diseases of Health Ministry, Tongji Hospital, Tongji Medical College, Huazhong University of Science and Technology, Wuhan, China; ^3^Department of Epidemiology and Biostatistics, School of Public Health, Tongji Medical College, Huazhong University of Science and Technology, Wuhan, China; ^4^Department of Infectious Diseases, Tongji Hospital, Tongji Medical College, Huazhong University of Science and Technology, Wuhan, China; ^5^Department of Laboratory Medicine, Tongji Hospital, Tongji Medical College, Huazhong University of Science and Technology, Wuhan, China

**Keywords:** SARS-CoV-2, COVID-19, nasopharyngeal swab, oropharyngeal swab, sensitivity, viral load

## Abstract

**Objective:** Detection of SARS-CoV-2 by oropharyngeal swabs (OPS) and nasopharyngeal swabs (NPS) is an essential method for coronavirus disease 2019 (COVID-19) management. It is not clear how detection rate, sensitivity, and the risk of exposure for medical providers differ in two sampling methods.

**Methods:** In this prospective study, 120 paired NPS and OPS specimens were collected from 120 inpatients with confirmed COVID-19. SARS-CoV-2 nucleic acid in swabs were detected by real-time RT-PCR. The SARS-CoV-2 detection rate, sensitivity, and viral load were analyzed with regards NPS and OPS. Sampling discomfort reported by patients was evaluated.

**Results:** The SARS-CoV-2 detection rate was significantly higher for NPS [46.7% (56/120)] than OPS [10.0% (12/120)] (*P* < 0.001). The sensitivity of NPS was also significantly higher than that of OPS (*P* < 0.001). At the time of sampling, the time of detectable SARS-CoV-2 had a longer median duration (25.0 vs. 20.5 days, respectively) and a longer maximum duration (41 vs. 39 days, respectively) in NPS than OPS. The mean cycle threshold (Ct) value of NPS (37.8, 95% CI: 37.0–38.6) was significantly lower than that of OPS (39.4, 95% CI: 38.9–39.8) by 1.6 (95% CI 1.0–2.2, *P* < 0.001), indicating that the SARS-CoV-2 load was significantly higher in NPS specimens than OPS. Patient discomfort was low in both sampling methods. During NPS sampling, patients were significantly less likely to have nausea and vomit.

**Conclusions:** NPS had significantly higher SARS-CoV-2 detection rate, sensitivity, and viral load than OPS. NPS could reduce droplets production during swabs. NPS should be recommended for diagnosing COVID-19 and monitoring SARS-CoV-2 load.

**Chinese Clinical Trial Registry, number**: ChiCTR2000029883.

## Introduction

Coronavirus disease 2019 (COVID-19) has developed into a devastating pandemic. As of April 20, 2020, there were 2,314,621 confirmed cases confirmed cases globally, and 157,847 people have lost their lives (1). This pathogen is a novel enveloped RNA beta coronavirus named severe acute respiratory syndrome coronavirus 2 (SARS-CoV-2) (2).

Detection of SARS-CoV-2 nucleic acid in upper respiratory specimens is essential for COVID-19 management, including diagnosis, risk assessment of transmission, and decisions regarding quarantine of patients. How to increase sensitivity of SARS-CoV-2 detection is key. To obtain upper respiratory specimens, medical providers usually use oropharyngeal swabs (OPS) and nasopharyngeal swabs (NPS) (3). However, it is unclear how the detection rate and sensitivity differ in the two sampling methods. Wang et al. (4) reported that the detection rate of SARS-CoV-2 was higher in nasal swabs [63% (5/8)] than in pharyngeal swabs [32% (126/398)]. Another small sample study analyzed 17 patients in early stages of COVID-19 and found that a higher viral load was detected in the nose than in the throat (5). Therefore, larger sample studies are needed to investigate that NPS specimens are more sensitive than OPS specimens for SARS-CoV-2 detection.

Meanwhile, during swab sampling, patients may feel uncomfortable and nauseous, causing them to cough, sneeze, and vomit. This may produce droplets and increase the risk of exposure for the medical providers (6). Currently, it is unclear how the risk differs in the two sampling methods.

In this prospective study, we investigated detection rate and sensitivity of SARS-CoV-2 in paired NPS and OPS from 120 confirmed COVID-19 patients. We also studied patient-reported discomfort level during sampling.

## Materials and Methods

### Study Design and Participants

In this prospective, single-center study, we recruited 120 laboratory-confirmed COVID-19 inpatients between February 15 and March 2, 2020, at Tongji Hospital, Tongji Medical College of Huazhong University of Science and Technology, in Wuhan, China. We excluded patients in critical conditions. Demographic data, comorbidities, symptoms, disease severity (7), imaging examinations, previous nucleic acid test results, and other laboratory findings on or close to the day of sampling were collected from electronic medical records using data collection forms. Another physician on our team reviewed the data independently. We obtained missing core data by direct communication with attending clinicians. None of the sampling operations affected the patients' normal treatment routines.

The studies involving human participants were reviewed and approved by the ethical committee of Tongji Hospital (file number TJ-IRB20200204). The requirement for written informed consent was waived by the ethics committee for this study, but all the participants provided their oral informed consent.

### Specimen Collection

Synthetic fiber swabs with plastic shafts and sampling tubes containing 3.5 mL viral transport medium were supplied by YOCON® (Beijing, China). Trained medical providers first labeled the tubes with patient information, then obtained paired NPS and OPS specimens. For NPS, patients were instructed to blow their noses; providers gently passed the swab into the posterior nasopharynx via the nostril, rotated for 10 s, and withdrew slowly (8). For OPS, providers wiped the pharyngeal tonsil and posterior pharynx with the swab, avoiding the tongue (9). Immediately after sampling, providers placed the swabs into transport media and tightened the tube cap. Swab samples were kept at 2–8°C and immediately submitted to the Clinical Lab of Tongji Hospital designated by the Chinese Center for Disease Control and Prevention.

Participants rated discomfort level experienced during the respective sampling. The questionnaires were collected from 103 patients. An arbitrary rating scale (1–4) was used with 1 being no discomfort and 4 being unbearable discomfort (9). Participants also rated specific symptoms during sampling, including rhinocnesmus, running nose, sneeze, cough, bleeding, nausea, vomit, and lachrymation, using a visual analog scale (VAS) with 0 being no feeling and 10 being the strongest feeling.

### Nucleic Acid Extraction and Real-Time RT-PCR for SARS-COV-2

After collection, RNA extraction and reverse transcription and polymerase chain reaction (RT-PCR) analysis were completed within 24 h according to the manufacturer′s instruction (DAAN Gene, Guangzhou, China). In brief, RNA was extracted from 200 μL of each sample with Viral RNA Isolation Kit, eluted in 50 μL of elution buffer, and used as the template for all assays. For real-time RT-PCR analysis, target genes including open reading frame (ORF1ab) and nucleocapsid protein (N) were simultaneously amplified and tested. Primer sequences for the ORF1ab gene were forward primer CCCTGTGGGTTTTACACTTAA; reverse primer ACGATTGTGCATCAGCTGA; and the probe 5′-FAM-CCGTCTGCGGTATGTGGAAAGGTTATGG-BHQ1-3′. Primer sequences for the N gene were forward primer GGGGAACTTCTCCTGCTAGAAT; reverse primer CAGACATTTTGCTCTCAAGCTG; and the probe 5′-VIC-TTGCTGCTGCTTGACAGATT-TAMRA-3′, which were recommended by the National Institute for Viral Disease Control and Prevention of China (http://ivdc.chinacdc.cn/kyjz/202001/t20200121_211337.html). The 25 μL RT-PCR reaction system contained 17 μL reaction mixture A, 3 μL reaction mixture B, and 5 μL RNA template. RT and PCR were performed under the following conditions of 50°C for 20 min, 95°C for 15 min, 45 cycles consisting of 94°C for 15 s, and 55°C for 45 s. The cut-off cycle threshold (Ct) value was 40 for both genes, and the Ct values of both genes were <40 was defined as positive. The Ct values were used as relative SARS-CoV-2 RNA expression with lower Ct values corresponding to higher viral copy numbers (4, 5).

### Statistical Analysis

Descriptive analyses on patient and clinical characteristics were presented as a median (interquartile range, IQR) or percentage (%). Primary results, including Ct values, patient-reported discomfort scores and detection rates, were reported as point estimates and 95% confidence intervals (95% CI). To investigate the diagnostic sensitivity of each method, we defined true positives as patients with positive SARS-CoV-2 result by at least one sampling method (10). McNemar's test or Wilcoxon signed rank test was used to compare the difference between the two sampling methods, unless otherwise indicated. Cohen's kappa statistics was used to determine the agreement of virus detection results between paired NPS and OPS. The statistical analysis was performed using the SAS software (version 9.4) with a two-sided significance threshold of *P* < 0.05.

## Results

### Clinical Characteristics of the Patients

This study included 120 hospitalized COVID-19 patients, 83/120 (69.2%) of which were in severe conditions (Table 1). At the time of sampling, the duration since symptom onset had a median of 27.0 days (IQR 23.0–31.5), ranging between 3 and 49 days. Most patients showed improvement during treatment: 108/120 (90.0%) were afebrile for at least 3 days, 115/120 (95.8%) had milder symptoms, and 98/105 (93.3%) had improved chest CT scans. Patients showed mostly normal laboratory findings. 61/120 patients (50.8%) already had one negative SARS-CoV-2 result by OPS, and they needed one more negative test result to meet discharge criteria (7).

**Table 1:** Baseline characteristics of the patients.

**Characteristics**	**All Patients**
**Age**	
Median (IQR)-yr	61.5 (47.5–69.0)
**Distribution-no./total no. (%)**	
≤40 yr	21/120 (17.5)
41–64 yr	53/120 (44.2)
≥65 yr	46/120 (38.3)
**Gender-no./total no. (%)**	
Female	53/120 (44.2)
Male	67/120 (55.8)
**Disease severity**^**§**^**-no./total no. (%)**	
Non-severe	37/120 (30.8)
Severe	83/120 (69.2)
**Comorbidities-no./total no. (%)**	47/120 (39.2)
Hypertension	36/120 (30.0)
Diabetes	20/120 (16.7)
Coronary heart disease	10/120 (8.3)
Cancer	6/120 (5.0)
Chronic respiratory diseases	2/120 (1.7)
**Initial symptoms-no./total no. (%)**	
Fever (≥37.3°C)	109/120 (90.8)
Cough	90/120 (75.0)
Dyspnea	56/120 (46.7)
Fatigue	42/120 (35.0)
Diarrhea	33/120 (27.5)
Chest tightness	31/120 (25.8)
Myalgia	29/120 (24.2)
Nausea or vomit	18/120 (15.0)
**Complete blood count-no./total no., median (IQR)**	
Leukocytes, per μL (reference range 3,500–9,500)	120/120, 6400 (5000-8200)
Neutrophil, per μL (reference range 1,800–6,300)	120/120, 4000 (3,000–5,700)
Lymphocyte, per μL (reference range 1,100–3,200)	120/120, 1600 (1,200–1,900)
Erythrocytes, per μL(reference range 3,800,000–5,800,000)	120/120, 4,100,000 (3,700,000–4,400,000)
Platelet, per μL (reference range 125,000–350,000)	120/120, 208,500 (167,800–274,000)
**Liver function-no./total no., median (IQR)**	
Alanine aminotransferase, U/L (reference range 0–33)	120/120, 27.0 (19.0–42.0)
Aspartate aminotransferase, U/L (reference range 0–32)	120/120, 21.0 (17.0–29.5)
**Renal function**-no./total no., median (IQR)^*^	
Urea, mmol/L (reference range 2.6–9.5)	109/120, 4.5 (3.6–5.8)
Creatinine, μmol/L (reference range 45–104)	110/120, 68.5 (58.0–87.0)
Inflammatory factors-no./total no., median (IQR)^*^	
Hs-CRP, mg/L (reference range 0–10)	104/120, 3.0 (1.2–7.0)
Procalcitonin, ng/mL (reference range 0–0.05)	112/120, 0.03 (0.02–0.06)
Interleukin-6, pg/mL (reference range 0–7)	94/120, 3.7 (1.5–9.9)
D-dimer, mg/L (reference range 0–0.5)	109/120, 0.8 (0.4–1.6)
Lactate dehydrogenase, U/L (reference range 135–225)	119/120, 201.0 (173.0–253.0)
Ferritin, μg/L (reference range 15–400)	95/120, 513.3 (295.3–848.2)
**Clinical outcomes at paired sampling-no./total no. (%) or median (IQR)**	
Days since onset of initial symptoms	27.0 (23.0–31.5)
Afebrile for at least 3 days (<37.3°C)	108/120 (90.0)
Symptoms improved	115/120 (95.8)
Chest CT improved	98/105 (93.3)
One more negative SARS-CoV-2 test for discharge^‡^	61/120 (50.8)

§ *The severe patients meeting any of the following criteria: respiratory distress (?30 breaths/ min);oxygen saturation ≤ 93% at rest; arterial partial pressure of oxygen (PaO_2_)/fraction of inspired oxygen (FiO_2_)≦ 300 mmHg (l mmHg = 0.133 kPa); obvious lesion progression within 24–48 h >50% of chest imaging (7)*.

* *no. /total no. denotes available number/total number because of some missing data of renal function, inflammatory factors*.

*no. /total no. denotes improved number/available number because of some missing data of chest CT*.

‡ The patients already had one negative SARS-CoV-2 test by OPS and needed one more negative test result to meet discharge criteria. Discharge criteria are afebrile for at least 3 days, respiratory symptoms significantly improved, improvement in the radiological abnormalities on chest radiograph or CT, and two consecutive negative SARS-CoV-2 tests more than 24 h apart (7).

*IQR, interquartile range; Hs-CRP, hypersensitive C-reactive protein; CT, computed tomography; SARS-CoV-2, severe acute respiratory syndrome coronaviruses 2*.

### NPS Had Higher Detection Rate of SARS-COV-2 Than OPS

To compare the detection rate of each method, we analyzed paired NPS and OPS specimens from 120 COVID-19 patients. Detection rate is the percentage of positive results in total samples. The detection rate of NPS is 46.7% (56/120), and the detection rate of OPS is 10.0% (12/120). NPS had a significantly higher detection rate of SARS-CoV-2 than OPS (*P* < 0.001, Kappa = 0.19 with 95% CI 0.07–0.31, Table 2).

**Table 2:** Detection of SARS-CoV-2 from NPS and OPS in all patients.

	**NPS positive**	**NPS negative**	**Total**
OPS positive	11	1	12
OPS negative	45	63	108
Total	56	64	120

*McNemar's test χ^2^ = 42.09, P < 0.001; Kappa = 0.19 (95% CI 0.07–0.31)*.

*Kappa: <0, poor; 0 to 0.2, slight; 0.21 to 0.4, fair; 0.41 to 0.6, moderate; 0.61 to 0.8, substantial; 0.81 to 1.0, almost perfect*.

*SARS-CoV-2, severe acute respiratory syndrome coronaviruses 2; NPS, nasopharyngeal swabs; OPS, oropharyngeal swabs*.

**Figure 1:**
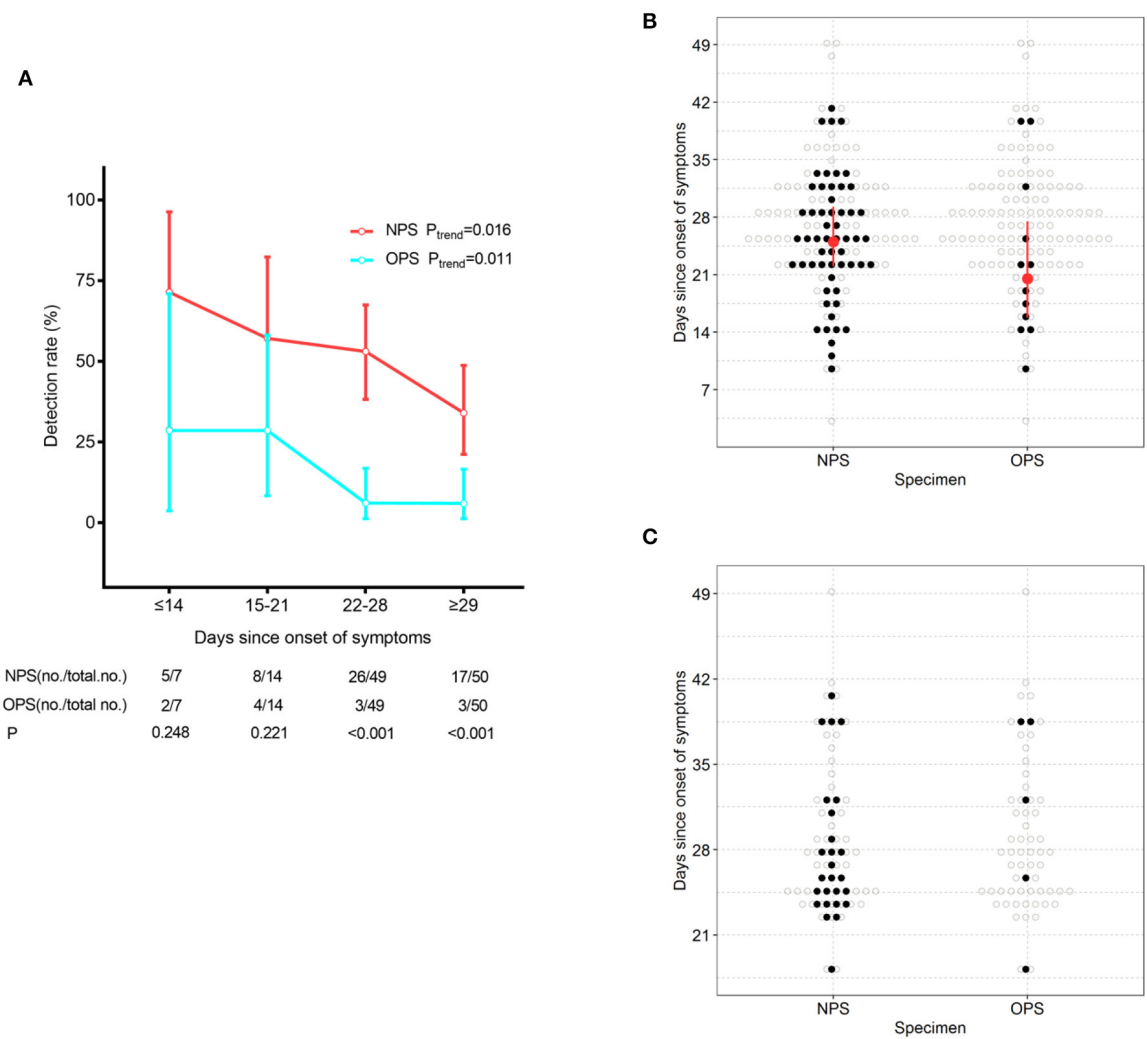
SARS-CoV-2 detection by NPS and OPS. **(A)** The detection rate (with 95% CI) of NPS and OPS with the development of the time course. **(B)** Time course (with IQR) of the detectable SARS-CoV-2 by paired NPS and OPS from 120 patients. **(C)** Time course of the detectable SARS-CoV-2 by paired NPS and OPS form 61 patients who needed one more negative SARS-CoV-2 result to meet the discharge criteria.

To understand whether treatment could confound the difference in detection rates, we stratified patients based on time since symptom onset. With the extension of the course of disease, positive detection rates of SARS-CoV-2 gradually decreased by both NPS and OPS (P_trend_ = 0.016 and 0.011, respectively, Figure 1A). A total of 21 days after symptom onset, NPS had a significantly higher detection rate than OPS (*P* < 0.001). Less than 21 days after symptom onset, although the detection rate of NPS was higher than that of OPS [≤14 days: 71.4% (5/7) vs. 28.6% (2/7), respectively; 15–21 days: 57.1% (8/14) vs. 28.6% (4/14), respectively], the difference was not significant.

At the time of sampling, the time of SARS-CoV-2 detection since symptom onset had a longer maximum duration (41 vs. 39 days, respectively) and a longer median duration (25.0 vs. 20.5 days, respectively) in NPS than OPS (Figure 1B).

Furthermore, we analyzed paired NPS and OPS specimens from 61 patients who needed one more negative SARS-CoV-2 result to meet the discharge criteria. 26/61 (42.6%) had positive NPS results, which did not meet the criteria and continued to be quarantined. Only 5/61 (8.2%) had positive OPS results and continued to be quarantined (Figure 1C).

### NPS Was More Diagnostically Sensitive in Detecting SARS-COV-2 Than OPS

To investigate the diagnostic sensitivity of each method, we identified 57 patients, who had tested positive for SARS-CoV-2 by either NPS or OPS, as true positives. Sensitivity is the percentage of true positives correctly identified by each method. The sensitivity of NPS was significantly higher than that of OPS [98.3% (56/57, 95% CI 94.8–100.0) vs. 21.1% (12/57, 95% CI 10.5–31.6), respectively, *P* < 0.001, Table S1].

Furthermore, to understand whether patient conditions could confound the difference in sensitivity, we stratified patients based on clinical characteristics and laboratory values. In all stratifications except febrile condition, the sensitivity of NPS was significantly higher than that of OPS (*P* < 0.05). In the seven febrile patients, there was no significant sensitivity difference between NPS and OPS, which may be explained by the small sample size.

### NPS Specimens Showed Higher SARS-COV-2 Load Than OPS

We then studied whether the difference in detection rate is caused by the difference in SARS-CoV-2 load in NPS and OPS specimens with regards to the duration since the symptom onset.

**Figure 2:**
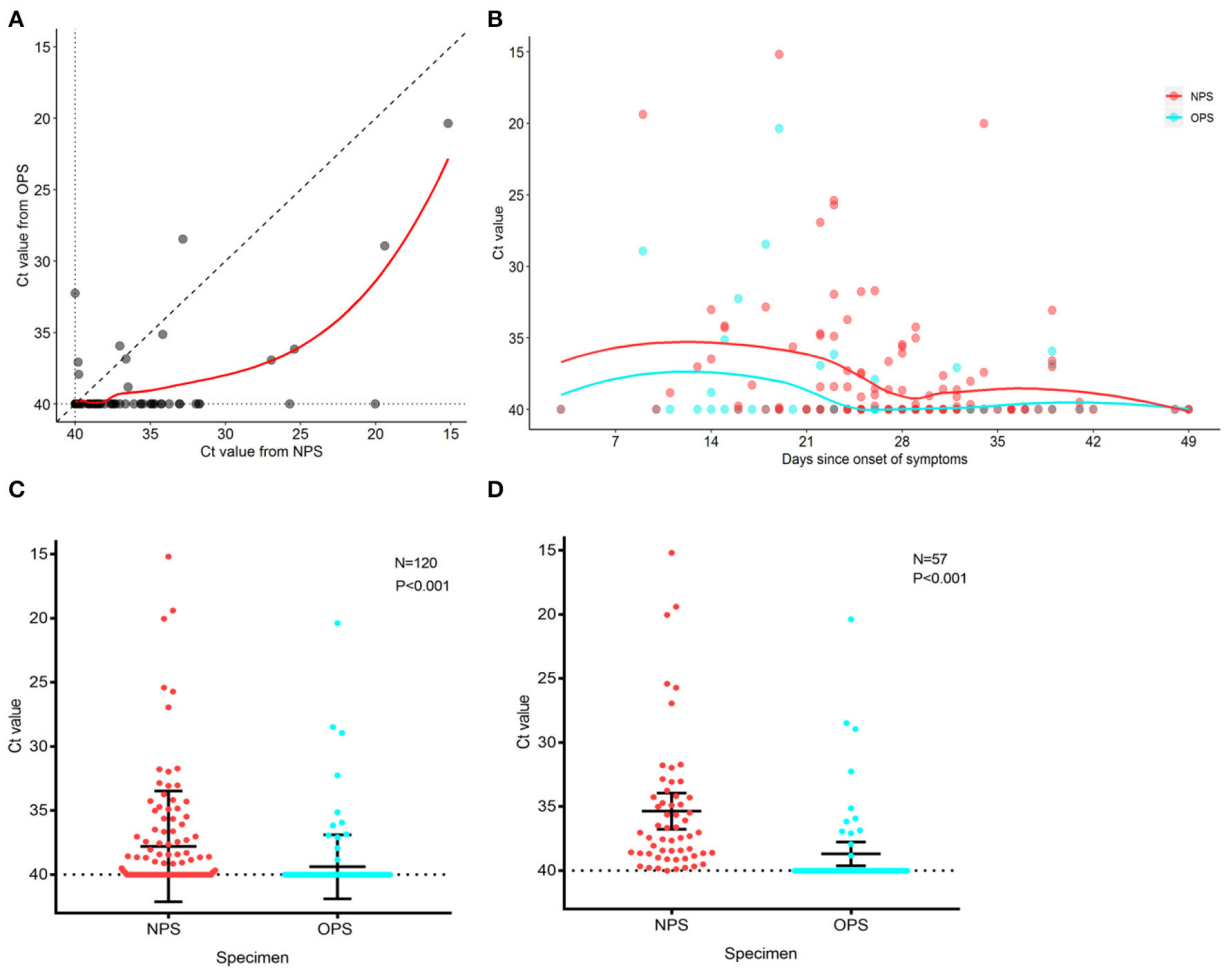
PCR cycle threshold (Ct) value detected in NPS and OPS specimens. **(A)** Comparison of Ct values of 120 paired NPS and OPS specimens. Each data point represents the Ct values of NPS and OPS from one patient. **(B)** Ct values for NPS and OPS during treatment. Solid curves represent the trend derived by locally weighted scatterplot smoothing method. **(C)** Comparison of Ct values (with 95% CI) of paired NPS and OPS from 120 patients. **(D)** Comparison of Ct values (with 95% CI) of paired NPS and OPS from 57 patients with positive SARS-CoV-2. A lower Ct value corresponds to a higher viral load.

To analyze the SARS-CoV-2 load of 120 paired specimens by real-time RT-PCR, we plotted NPS cycle threshold (Ct) values against OPS Ct values (Figure 2A). The Ct values were used as relative SARS-CoV-2 RNA expression with lower Ct values corresponding to higher viral copy numbers (4, 5). The mean Ct value of NPS (37.8, 95% CI 37.0–38.6) was significantly lower than that of OPS (39.4, 95% CI 38.9–39.8) by 1.6 (95% CI 1.0–2.2, *P* < 0.001) (Figure 2C), indicating a significantly higher viral load in NPS specimens. During treatment, NPS and OPS Ct values both increased, NPS Ct values were consistently lower than OPS Ct values (Figure 2B).

In 57 patients with positive SARS-CoV-2 by either NPS or OPS, 52/57 (91.2%) had NPS Ct value lower than OPS Ct value (Figure 2A). In other words, NPS specimens from true positive patients had a higher viral load than OPS. The mean Ct value of NPS (35.3, 95% CI 33.9–36.8) was significantly lower than that of OPS (38.7, 95% CI 37.7–39.6) by 3.3 (95% CI 2.2–4.5, *P* < 0.001), indicating that the viral load was 10 times higher in NPS specimens than OPS (95% CI 4.6–22.6) (Figure 2D).

### Evaluation of Patient-Reported Discomfort Levels and Symptoms During Sampling

**Figure 3:**
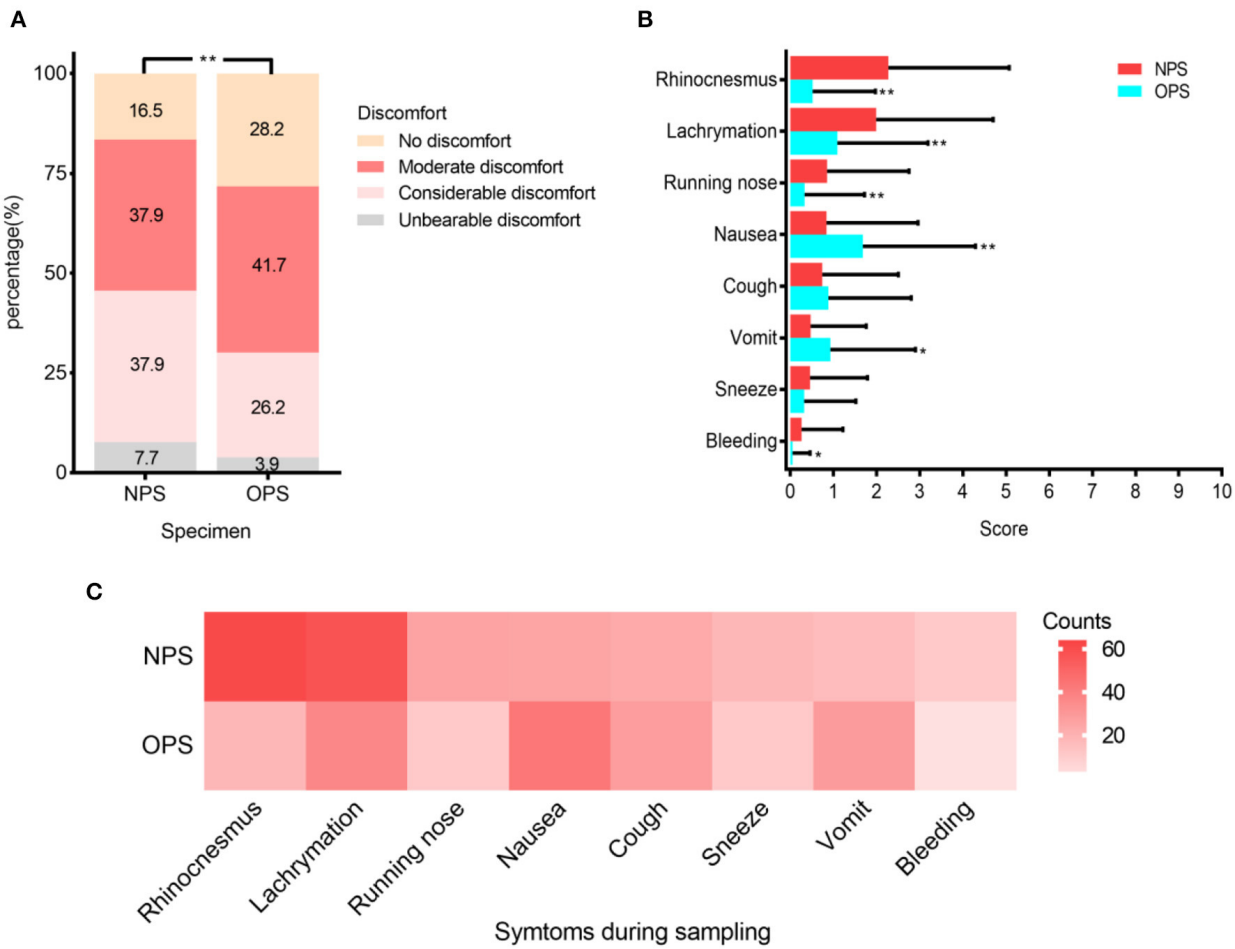
Patient discomfort levels during NPS and OPS sampling (*N* = 103). **(A)** Percentage of four varying discomfort levels reported by patients during swab sampling. **(B)** Mean scores (with standard deviation) of symptoms during sampling. **(C)** Frequency of each symptom during sampling. *P < 0.01, **P < 0.001.

To study patient discomfort level during sampling and possibility of droplets production, we analyzed questionnaires from 103 patients. Patients reported significantly higher overall discomfort levels when taking NPS (*P* < 0.001) (Figure 3A). The reported discomfort levels caused by each symptom, including rhinocenesmus, lachrymation, running nose, nausea, coughing, vomit, sneezing, and bleeding, were low in both NPS and OPS, with average scores all <3 out 10 (Figure 3B). When taking OPS, patients were significantly more likely to have nausea and vomit (*P* < 0.01) than NPS. Although patients coughed (23 vs. 28 patients, respectively) and sneezed (18 vs. 11 patients, respectively) during NPS and OPS, the difference was not significant (Figure 3C).

## Discussion

This prospective study analyzed paired NPS and OPS specimens for SARS-CoV-2 detection by real-time RT-PCR in 120 inpatients with laboratory-confirmed COVID-19. We found that NPS was more sensitive to detecting SARS-CoV-2 than OPS. The SARS-CoV-2 load was higher in NPS specimens. As the patients' conditions improved, viral load in the upper respiratory tract decreased but could be detected for longer time in NPS specimens. Patient discomfort was low in both sampling methods. During NPS sampling, patients had significantly less nausea and vomit, which could lead to reduced droplet production, thus decreasing the risk of exposure for medical providers.

The SARS-CoV-2 detection rate of pharyngeal swabs is low [32% (126/398)] (4). For influenza B and A, diagnostic sensitivity of NPS [78% (25/32)] was higher than OPS [63% (20/32)] (11). It is unclear how the detection sensitivity for SARS-CoV-2 differs in NPS and OPS. We found that in 120 COVID-19 patients, NPS had a significantly higher SARS-CoV-2 detection rate than OPS (46.7% (56/120) and 10.0% (12/120), respectively) (Table 2). The detection rate for SARS-CoV-2 was lower than influenza in both sampling methods. Most patients in this study were in recovery (Table 1), so SARS-CoV-2 shedding could explain the low detection rate, and 63/120 (52.5%) patients presented negative SARS-CoV-2. With the extension of the time course and the progressed treatment, detection rates of NPS and OPS both decreased (Figure 1A). However, compared to the OPS samples obtained at the same time, NPS consistently had higher detection rate, especially 21 days after symptoms onset (Figures 1A,B).

Sixty-one patients who needed one more negative SARS-CoV-2 result to meet the discharge criteria (7), took paired NPS and OPS. A total of 26/61 (42.6%) exhibited positive NPS results and continued to be quarantined, but only 5/61 (8.2%) exhibited positive OPS results and were required to be quarantined (Figure 1C). This finding suggested that there were false-negative results in OPS specimens. In other words, if providers only analyzed OPS specimens, patients with positive SARS-CoV-2 could be mistakenly released from quarantine, increasing the risk of transmission to the public.

Zhou et al. (3) found that in COVID-19 survivors, the duration of viral shredding in OPS had a median of 20 and a maximum of 37 days. We found that at the time of sampling, the detectable SARS-CoV-2 in OPS persisted for a median of 20.5 days and a maximum of 39 days, which was consistent with Zhou's findings (Figure 1B). However, compared to OPS, detectable SARS-CoV-2 in NPS had a longer median duration (25 days) and maximum duration (41 days, Figure 1B). The maximum duration of viral shedding in NPS was longer than what was reported by Young et al. (12), who suggested that the duration of viral shedding from nasopharyngeal aspirates could persist up to at least 24 days after symptom onset. These findings indicated that NPS could detect SARS-CoV-2 for a longer duration after symptom onset.

To investigate the diagnostic sensitivity of each method, we identified the 57 patients who exhibited positive SARS-CoV-2 in either NPS or OPS as true positives. NPS showed significantly higher sensitivity than OPS in 57 paired NPS and OPS specimens [56/57 (98.3%) and 12/57 (21.1%), respectively] (Table S1). The sensitivity difference was not affected by clinical characteristics or laboratory findings, except for afebrile condition. This result suggested that NPS was more diagnostically accurate than OPS.

All patients received treatment after disease confirmed. We found that with the extension of the time course and the progressed treatment, while the SARS-CoV-2 load decreased, NPS specimens had a consistently higher viral load than OPS specimens (Figure 2B). Zou et al. (5) also reported that the SARS-CoV-2 load was significantly higher in nasal samples than in throat samples from 17 patients in early stages of COVID-19. We found that the viral load in NPS specimens was 10 times higher than OPS, in 57 patients with positive results by either NPS or OPS (Figures 2A,C). Altogether, the reasons that NPS had a higher virus load and higher sensitivity than OPS could be: (1) the amount of virus was higher in the nasopharynx than the oropharynx after SARS-CoV-2 infection; (2) NPS had a larger contact surface area with the nasopharynx, leading to more of the virus being collected.

During sampling, patients could produce droplets, thus increasing the risk of exposure for medical providers (6). We evaluated the patients' discomfort levels and droplet-producing symptoms, such as nausea, vomiting, coughing, and sneezing. The discomfort caused by the two sampling methods was similar to other respiratory virus sampling, and the symptoms were generally mild (Figures 3A,B) (9). Patients were significantly more likely to have nausea and vomit during OPS than NPS. Although the differences in coughing and sneezing during NPS and OPS sampling were not significant, the frequencies of coughing were lower in NPS than OPS (23 vs. 28 times, respectively). These results suggested that NPS sampling may be associated with less droplet production. Additionally, droplets produced during NPS and risk of exposure can be easily reduced, if medical providers stand next to the patient instead of face-to-face and cover the patient's mouth with a face mask (6).

Our study has limitations. First, most patients in this study were in recovery. The median duration since symptom onset was 27 days. Patients could have viral shedding, and so detection rates may not accurately reflect diagnostic sensitivity. Second, we could not quantify droplets produced due to equipment limitations. Instead, we used symptoms during sampling to represent the possibility of droplets produced.

In summary, we found that NPS was more sensitive for SARS-CoV-2 detection than OPS. NPS specimens had higher SARS-CoV-2 load than OPS specimens. NPS could reduce droplets production during swabs, especially when combined with other approaches. NPS should be recommended for diagnosing COVID-19 and monitoring SARS-CoV-2 load.

## Data Availability Statement

The raw data supporting the conclusions of this article will be made available by the authors, without undue reservation.

## Ethics Statement

The studies involving human participants were reviewed and approved by the Ethical Committee of Tongji Hospital, Tongji Medical College, Huazhong University of Science and Technology. Written informed consent from the participants' legal guardian/next of kin was not required to participate in this study in accordance with the national legislation and the institutional requirements.

## Author Contributions

HW, QL, and SX contributed to conception and design of the study. QL and SX organized the database. HW, MZ, MY, KL, and DX carried out sample collection and data acquisition. YL and FW performed the laboratory sample analysis. JH, YX, JY, and PY performed the statistical analysis. SX wrote the first draft of the manuscript. HW, QL, and JH wrote sections of the manuscript. All authors approved the final manuscript as submitted and agreed to be accountable for all aspects of the work.

## Conflict of Interest

The authors declare that the research was conducted in the absence of any commercial or financial relationships that could be construed as a potential conflict of interest.
