# Supplementary material for: Nasopharyngeal Swabs Are More Sensitive Than Oropharyngeal Swabs for COVID-19 Diagnosis and Monitoring the SARS-CoV-2 Load
Source: Front Med (Lausanne). 2020 Jun 18;7:334. doi: 10.3389/fmed.2020.00334 (PMC7314917; doi:10.3389/fmed.2020.00334)
Supplement: Supplementary file 1 [file Table_1.DOCX]

Supplementary Material

# Table S1. Diagnostic accuracies of NPS and OPS according to patient characteristics.

|  |  |  |  | **NPS** |  |  | **OPS** |  |  |
| --- | --- | --- | --- | --- | --- | --- | --- | --- | --- |
|  |  |  | **No.** |  | **Negative** | **No.** |  | **Negative** |  |
| **Characteristics** | **Total** | **No.** | **identified** | **Sensitivity^‡^** | **predictive** | **identified** | **Sensitivity^‡^** | **predictive** | **P value^¶^** |
|  | **(n=120)** | **positive** | **(n=56)^†^** | **(95% CI)** | **value^§^** | **(n=12)^†^** | **(95% CI)** | **value^§^** |  |
|  |  | **(n=57)^*^** |  |  | **(95% CI)** |  |  | **(95% CI)** |  |
| **Total** | 120 | 57 | 56 | 98.3(94.8-100.0) | 98.4(95.4-100.0) | 12 | 21.1(10.5-31.6) | 58.3(49.0-67.6) | <0.001^#^ |
| **Days since onset of symptoms** | | | | | | | | | |
| ≤21 | 21 | 14 | 13 | 92.9(79.4-100.0) | 87.5(64.6-100.0) | 6 | 42.9(16.9-68.8) | 46.7(21.4-71.9) | 0.04 |
| 22-28 | 49 | 26 | 26 | 100.0(.-.) | 100.0(.-.) | 3 | 11.5(0-23.8) | 50.0(35.6-64.5) | <0.001 |
| ≥29 | 50 | 17 | 17 | 100.0(.-.) | 100.0(.-.) | 3 | 17.7(0-35.8) | 70.2(57.1-83.3) | <0.001 |
| **Disease severity** | | | | | | | | | |
| Severe | 83 | 41 | 40 | 97.6(92.8-100.0) | 97.7(92.8-100.0) | 11 | 26.8(13.3-40.4) | 58.3(47.0-69.7) | <0.001 |
| Non-severe | 37 | 16 | 16 | 100.0(.-.) | 100.0(.-.) | 1 | 6.3(0-18.1) | 58.3(42.2-74.4) | <0.001 |
| **Comorbidities** | | | | | | | | | |
| Comorbidities | 48 | 26 | 25 | 96.2(88.8-100.0) | 95.7(87.3-100.0) | 8 | 30.8(13.0-48.5) | 55.0(39.6-70.4) | <0.001 |
| Non- comorbidities | 72 | 31 | 31 | 100.0(.-.) | 100.0(.-.) | 4 | 12.9(1.1-24.7) | 60.3(48.7-71.9) | <0.001 |
| **Fever within three days before sampling (≥ 37.3℃)** | | | | | | | | | |
| Fever | 12 | 7 | 7 | 100.0(.-.) | 100.0(.-.) | 2 | 28.6(0-62.0) | 100.0(.-.) | 0.06 |
| Non-fever | 108 | 50 | 49 | 98.0(94.1-100.0) | 98.3(95.0-100.0) | 10 | 20.0(8.9-31.1) | 59.2(49.5-68.9) | <0.001^#^ |
| **Chest CT improved (missing=15)** | | | | | | | | | |
| Non-improved | 7 | 1 | 1 | 100.0(.-.) | 100.0(.-.) | 1 | 100.0(.-.) | 100.0(.-.) | NA |
| Improved | 98 | 46 | 45 | 97.8(93.6-100.0) | 98.1(94.5-100.0) | 6 | 13.0(3.3-22.8) | 56.5(46.4-66.7) | <0.001 |
| **IL-6 (missing=26)** | | | | | | | | | |
| Increased | 30 | 15 | 15 | 100.0(.-.) | 100.0(.-.) | 4 | 26.7(4.3-49.1) | 57.7(38.7-76.7) | 0.001 |
| Normal | 64 | 30 | 30 | 100.0(.-.) | 100.0(.-.) | 6 | 20.0(5.7-34.3) | 58.6(46.0-71.3) | <0.001 |
| **D-dimer (missing=11)** | | | | | | | | | |
| Increased | 71 | 38 | 38 | 100.0(.-.) | 100.0(.-.) | 8 | 21.1(8.1-34.1) | 52.4(40.1-64.7) | <0.001 |
| Normal | 38 | 12 | 11 | 91.7(76.0-100.0) | 96.3(89.2-100.0) | 3 | 25.0(0.5-49.5) | 74.3(59.8-88.8) | 0.02 |
| **Lactate dehydrogenase (missing=1)** | | | | | | | | | |
| Increased | 48 | 27 | 26 | 96.3(89.2-100.0) | 95.5(86.8-100.0) | 9 | 33.3(15.6-51.1) | 53.9(38.2-69.5) | <0.001 |
| Normal | 71 | 30 | 30 | 100.0(.-.) | 100.0(.-.) | 3 | 10.0(0-20.7) | 60.3(48.7-71.9) | <0.001 |
| **Ferritin (missing=25)** | | | | | | | | | |
| Increased | 78 | 36 | 36 | 100.0(.-.) | 100.0(.-.) | 9 | 25.0(10.9-39.1) | 60.9(49.4-72.4) | <0.001 |
| Normal | 17 | 8 | 8 | 100.0(.-.) | 100.0(.-.) | 1 | 12.5(0-35.4) | 53.3(28.1-78.6) | 0.02 |

^*^ The number of paired swabs identified as SARS-CoV-2 positive by NPS or NPS.

^†^ The number of swabs identified as SARS-CoV-2 positive by NPS (or OPS).

^‡^ The number of swabs identified positive by NPS (or OPS) / the number of paired swabs identified as SARS-CoV-2 positive by NPS or NPS.

*^§^* The number of swabs identified negative by both NPS and OPS / the number of swabs identified as SARS-CoV-2 negative by NPS (or OPS).

^¶^ Comparison of sensitivity between NPS and OPS, McNemar’s test with exact binomial confidence limits, ^#^ means McNemar’s test.

NPS, nasopharyngeal swabs. OPS, oropharyngeal swabs. CI, confidence interval. NA, not applicable.
